# Do cats with a cranial cruciate ligament injury and osteoarthritis demonstrate a different gait pattern and behaviour compared to sound cats?

**DOI:** 10.1186/s13028-016-0248-x

**Published:** 2016-10-20

**Authors:** Sarah Stadig, B. Duncan X. Lascelles, Anna Bergh

**Affiliations:** 1 Department of Clinical Sciences, Faculty of Veterinary Medicine and Animal Husbandry, Swedish University of Agricultural Sciences, PO Box 234, S532 23 Skara, Sweden; 2 Comparative Pain Research, Department of Clinical Sciences, College of Veterinary Medicine, North Carolina State University, Raleigh, NC 27606 USA; 3 Center for Pain Research and Innovation, School of Dentistry, University of North Carolina, Chapel Hill, NC USA; 4 Department of Anatomy, Physiology and Biochemistry, Faculty of Veterinary Medicine and Animal Husbandry, Swedish University of Agricultural Sciences, PO Box 7011, S750 07 Uppsala, Sweden

**Keywords:** Feline, Cranial cruciate ligament, Pain questionnaire, Owner assessment, Pressure mat technique

## Abstract

**Background:**

Osteoarthritis (OA) is a common cause of chronic pain and dysfunction in older cats. The majority of cats with OA do not show signs of overt lameness, yet cats with orthopaedic disease are known to redistribute their body weight from the affected limb. OA can cause changes in the cat’s behaviour, which is often misinterpreted as signs of aging. The aim of the present study was to investigate if cats with a previous cranial cruciate ligament (CCL) injury perform differently on the pressure mat and exhibit different behaviour compared to sound cats according to the owner´s subjective assessment. Ten cats with a previous CCL injury were assessed with a pressure mat system and their owners were asked to complete an assessment questionnaire. The results were compared to those of 15 sound cats, matched to have the same weight and body condition score.

**Results:**

The front/hind limb index for peak vertical force (PVF) was significantly higher for CCL cats, and there was a decreased PVF and vertical impulse (VI) on the affected hindlimb compared to the unaffected one. The results indicate that cats with a previous CCL injury put less weight, on the affected hindlimb but for a longer time. There was a significantly higher owner assessment questionnaire score for the group of cats with CCL injury compared to sound cats.

**Conclusions:**

Cats with a previous CCL injury have a different gait pattern compared to sound cats and a different behaviour according to owner subjective assessment. It is of great importance that further studies are performed to investigate the long term effects of CCL injury as a cause of pain and physical dysfunction, and its role in the development of OA in cats. Improved assessment tools for chronic pain caused by OA in cats are needed, both to facilitate diagnosis and to evaluate pain-relieving treatment.

## Background

Appendicular joint osteoarthritis (OA) is a frequently occurring disease in older cats [1–5]. As in other mammals, it causes chronic pain and physical disability. It is a challenge to diagnose OA in cats since they appear to have an innate ability to disguise injury and disease, particularly in unfamiliar surroundings, such as the clinical examination room [6, 7]. Furthermore, the cat demonstrates non-specific clinical signs, such as being less active [1, 3, 8–10]. These findings are often misinterpreted as signs of normal aging.

Currently, the diagnosis of OA in the cat is based on a combination of information from the cat owner, the cat´s medical history, clinical examination and radiography. The results from radiography are not always consistent with the clinical findings from the orthopaedic examination [2, 11, 12]. Despite this inconsistency, radiographic imaging is still considered the primary diagnostic instrument for suspected OA in cats [3, 13, 14].

The majority of cats with OA do not show signs of overt lameness [2, 3, 5, 11], yet orthopaedic disease is known to cause a redistribution of body weight from the affected limb [15–17]. The lack of overt signs of lameness in the majority of cats may be due to the fact that there is multiple limb involvement in the majority of cases. Despite this fact, studies have shown that kinetic data generated using a pressure mat system can detect cats with an asymmetric gait pattern that may be associated with appendicular OA [18, 19]. In addition, the information that the cat owner provides regarding behavioural changes has been shown to make an important contribution to the diagnosis of OA [8, 9, 20–22].

Most cases of feline OA appear to be primary or idiopathic and the actual cause or aetiology can only be established in a small number of cases [1, 2]. Hardie et al. suggested that observed cases of OA were likely secondary to undetermined previous factors such as dysplasia, chronic low grade trauma or malarticulation [3]. However, these conclusions appear to be based on the aetiology of OA in dogs and may not be applicable in the cat. For example, recent information in cats has indicated that they do not appear to suffer from fragmented coronoid process, the most common cause of elbow OA in dogs [23]. In human patients, obesity is regarded as a predisposing factor for OA [24, 25]. This has not been established in the cat, although there is one study showing that heavier cats were more likely to be taken to the veterinarian because of lameness [26].

In the feline stifle joint, injury to the cranial cruciate ligament (CCL) has been shown to lead to OA. Transection of the CCL in cats has been used as a model to study the mechanisms behind traumatic joint injury and the degenerative response, eventually leading to OA. The altered joint mechanics have been shown to cause adaptive changes in the articular cartilage [27, 28], the periarticular bone [29], the muscle mass [27] and the vertical ground reaction forces of the affected hindlimb [27, 30].

However, little is known concerning the aetiology, clinical features and the outcome of treatment in feline CCL injury. Most published information on feline CCL disease is in the form of case reports describing various surgical stabilization techniques [31–39]. Some authors suggest two aetiologically different groups of CCL injuries, traumatic and degenerative [40, 41] and discuss whether obesity is a cause or a result of an injury. Furthermore, there is an ongoing discussion regarding the relative roles of obesity and CCL injury in the development of stifle OA. Consequently, there is a need for additional studies on cats with CCL injury and eventually secondary OA.

The aim of the present study was to investigate if cats with previous CCL injury perform differently on the pressure mat compared to sound cats, and whether they show behavioural changes according to an owner assessment questionnaire. The hypotheses were:

1. Cats with a previous CCL injury have more asymmetrical front/hind symmetry indices for peak vertical force (PVF) and vertical impulse (VI), compared to sound cats.
2. Cats with a previous CCL injury have a decreased PVF and VI on the affected hindlimb compared to the unaffected hindlimb.
3. Cats with a previous CCL injury have a different way of distributing the pressure under the paws, compared to sound cats.
4. Cats with a previous CCL injury have a different behaviour, compared to sound cats.

## Methods

The participating CCL cats were privately owned pet cats recruited from the patient data base at the local animal hospital. The sound cats were selected from a previous dataset [42]. The study was approved by the local Ethical Review Board on Animal Experiments (no. C23/15). Prior to inclusion each cat owner signed an informed client consent form.

### Animals

#### Cats with previous CCL injury

The inclusion criteria for the study were as follows:

- Age between 1 and 12 years old, of either sex, and any breed other than Scottish fold, with a previous diagnosis of unilateral CCL injury.
- Have results from haematology and serum biochemistry analyses (haemoglobin, albumin, total protein, alanine transaminase (ALT), alkaline phosphatase (ALP), urea and creatinine) that are within the reference intervals of a healthy reference population of cats.

Exclusion criteria were as follows:

- Cats with gait disturbances caused by diseases other than a previous CCL injury in the affected stifle joint.
- Palpatory findings on clinical and orthopaedic examination of the musculoskeletal system of a larger magnitude then that from the affected stifle joint.
- Cats that were on any treatment for OA or other disease, with either a registered pharmaceutical or a supplement or a diet containing substances with alleged effect on OA.

#### Normal controls

Fifteen sound cats were selected from a previous dataset [42] to match the weight and body condition score of the cats with previous CCL injury. They were determined as sound based on their medical history, that is, without any disease or injury that could interfere with the study, on the clinical and orthopaedic examination, and from the results of the owner assessment questionnaire and a symmetrical gait based on pressure mat registrations [42].

### Outcome measures

One owner-administered clinical metrology instrument (referred to as owner assessment questionnaire) and gait analysis from a pressure mat was used as outcome measures.

#### Owner assessment questionnaire

The owner filled out Bennet and Morton’s owner assessment questionnaire [8, 22]. It is designed to identify changes within four behavioural domains (mobility, activity, grooming and temperament). The cat-owner marks whether the behaviour is normal or abnormal. If it is abnormal the owner is asked to rate the severity of the problem from 1 to 10. The owner is also asked to rate the overall severity of the problem from 1 (mild) to 10 (severe). The maximum score is 50, indicating an abnormal behaviour.

#### Pressure mat technique

The kinetic data were collected using a pressure sensitive walkway (Walkway High Resolution HRV4; Tekscan, South Boston, Massachusetts, USA). The portable mat measures 1.95 × 0.45 m and consists of a low profile floor mat (0.57 cm thick). The walkway was connected to a laptop computer (Siemens Fujitsu Lifebook, Hewlett Packard EliteBook) and data were analyzed using specific software provided by the manufacturer (Walkway 7.02). The mat was placed against a wall and transparent plexiglas screens, each 1.0 m long, were placed along the other side of the mat. The walkway was covered with a 1.0 mm thick plastic mat to avoid the slick surface, extending 0.3 m on either side of the end-/starting points for the sensors. The actual end-points of the walkway were demarcated with white tape. Prior to commencing data acquisition the walkway sensors were equilibrated and calibrated as recommended by the manufacturer. The data acquisition parameter was set to a frequency of 60 Hz, and each data movie was accompanied by a simultaneous video capture of the pass.

### Study protocol

The study was performed in a quiet examination room used for feline gait analyses. The owners completed the assessment questionnaire in the same room. The cat was weighed on an electronic scale and allowed to acclimatize to the surroundings for 5–10 min before walking on the mat. The cat was encouraged to walk on the mat by being called, using toys or treats, or by placing the transport carrier at the end of the mat. Data collection continued until five valid trials were attained. A trial was considered valid when the cat walked in a straight line, at a visually even pace and with the head facing straight forward.

A complete clinical and orthopaedic examination of the axial and appendicular skeleton, including evaluation of muscle symmetry, was carried out on each cat, after pressure mat data collection. It was performed by the same veterinarian (SS) each time, who also evaluated the cat´s body condition score (BSC) according to a 5-point system [43] and a 9-point system [44]. The evaluation was made by palpating the ribs, lumbar vertebra and abdominal fat pad according to the written instructions for each scoring system. The cat was also visually inspected from above and from the side to evaluate its contour and absence or presence of a waist. This was then compared to the illustrations on each scoring system. The evaluator calibrated herself against both scoring systems every day they were used. The cats were screened for neurological conditions that could cause pain, gait abnormalities or other symptoms. Every joint was palpated for periarticular thickening, joint effusion and pain response to palpation. The joints were also examined for range of motion and joint stability. The results from the examination of the joints is presented (Table 1) as the cumulative assessment of each joint, graded as mild, moderate or severe.

**Table 1:** Clinical characteristics of cats with previous cranial cruciate ligament injury

Cat no	Age	Weight at exam	Time since injury (months)	Treatment	Visual gait assessment	Cumulative assessment joint examination	Summary of radiographic findings
1	12	5	30	L: S	Mildly stiff gait hind	R elbow mild changes, L stifle severe, hip bilateral and R stifle moderate changes	R stifle* mild to moderate OA, R tarsometatarsal joint suspected OA, both coxofemoral joints mild to moderate OA, R shoulder joint moderate to severe OA, L shoulder joint mild OA
2	11	4.6	88	R: S	Mildly increased hyperextension stifle joints bilateral	R stifle moderate changes, L stifle mild changes	R stifle moderate OA, L stifle minute changes, possible OA, but uncertain finding
3	9	4.5	22	R: S	Moves with increased width between hindlimbs	L stifle moderate changes, R stifle mild changes	L stifle moderate to severe OA, R stifle mild OA
4	12	3.5	2	L: S	Mild hyperextension L stifle joint, 1° lameness LH	L stifle moderate changes	Only presurgical radiographs
5	8	5.9	48	L: C	Mildly stiff gait hind, 1° lameness LH	L stifle moderate changes	L stifle moderate OA
6	10	5.4	3	R: C	Mildly stiff gait hind	L stifle moderate changes, R stifle mild changes	L and R stifle mild to moderate OA
7	8	5.6	4	L: C	Mildly stiff gait hind	R stifle mild changes	R stifle soft tissue findings, no radiographic OA
8	10	5.3	18	L: S	LH: shorter stride and mild hyperflexion stifle joint	L stifle moderate changes	R stifle moderate to severe OA, L stifle small uncertain finding, not OA, R coxofemoral joint moderate OA
9	8	4.2	24	L: S	LH mild hyperflexion stifle joint, 1° lameness LH	L stifle moderate changes	L stifle severe OA
10	7	6.8	7	L: C	Mildly stiff gait hind	L stifle moderate changes	L stifle moderate OA

Grading of findings: mild, moderate, severe. Lameness graded 0–5 according to American Association of Equine Practitioners (AAEP)

*C* conservative treatment,* L* left, *LH* left hind, *OA* osteoarthritis, *R* right, *S* surgical treatment

*Left stifle was not radiographed

After this procedure was concluded, a blood sample was taken from the cats. If the haematology and serum biochemistry analyses were normal, the cats were sedated with a combination of medetomidine and butorphanol. The joints that were found to be affected on the orthopaedic examination were radiographed. Sedation was reversed with atipamezole. The sound cats went through the same procedure as the cats with previous CCL injury, apart from the blood sampling and radiographic examination.

### Data analyses

**Fig. 1:**
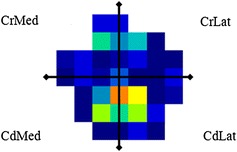
Distribution of the vertical force during one strike, right front paw. The paw print was divided into four equally sized areas for analysis. Craniolateral (CrLat), Caudomedial (CdMed), Craniolateral (CrLat), Craniomedial (CrMed)

The symmetry indices for front/hind PVF were calculated by dividing the average PVF of the two front feet by the PVF of the two hind feet. The left/right symmetry indices were calculate (with PVF left hind/right hind as an example) by taking the average of the PVF of all left hind foot stances, divided by the PVF of all right hind foot stances. This was done according to the manufacturer´s instructions by the software program (Walkway 7.02). The data was evaluated for normal distribution using normal probability plots for the residuals. Data was log-transformed in some cases where skewness was detected from residual plots. The kinetic variables calculated were peak vertical force (PVF) and vertical impulse (VI). These were expressed as a percentage of total bodyweight (%BW) with approximate 95 % confidence intervals. Difference in gait parameters was investigated using the freely-available statistical software, R [45] mixed linear models, with health status as fixed factor and cat as random factor. Since there was no statistical difference depending on whether the cats had their CCL injury on the left or right hind, these data were analysed together. When analysing the distribution of the vertical forces within a paw, measurements of PVF (%BW) and VI (%BW*sec) were obtained by dividing the paw print into four equally sized areas: craniolateral, craniomedial, caudolateral and caudomedial (Fig. 1, for further details see [42]). Data regarding age, weight and BCS are presented as mean (±standard deviation). ANOVA was used to compare inter-cat variability. The correlation between the parameters age, weight and BCS was analysed using intraclass correlation-coefficient [46]. Difference in Bennett’s questionnaire was tested using Mann–Whitney’s test. The level of significance was set at P < 0.05.

## Results

### Clinical characteristics

The clinical characteristics of the cats with previous CCL injury are presented in Table 1. Of the 10 cats, 70 % were domestic shorthair and 30 % were purebred (one British shorthair, one European shorthair and one Siberian cat). Six cats were male and four female; all were neutered or spayed. The average age was 9.5 (±1.8) years and the mean weight 5.1 (±0.9) kg. They had a BCS with a mean of 3.8 (±0.4) on a 5-point system [43] and a BCS of 6.6 (± 0.8) on a 9-point system [44]. All cats with previous CCL injury had palpable periarticular thickening of the affected stifle joint. A decreased range of motion (8/10), and muscle atrophy proximally of the affected stifle joint (8/10), were common findings on clinical examination. Few cats had joint crepitus (1/10) or palpable joint effusion (2/10). Most of the cats showed signs of pain on manipulation of the affected stifle joint, either on palpation of the actual joint (3/10) or at the extremes of the range of motion (8/10).

Of the 15 sound cats, 60 % were domestic shorthair and 40 % were purebred, the latter consisting of one Somali, two Ragdolls, two Burmese and one Norwegian forest cat. Nine were neutered males, four were spayed females and two were intact females. The average age was 5.9 (±3.3) years and the mean weight was 4.8 (±0.9) kg. They had a body condition score (BCS) of 3.7 ± 0.5 on a 5-point system [43] and a BCS of 6.5 (±0.9) on a 9-point system [44]. The cats were matched regarding BCS in order to avoid the possibility that the findings from the pressure mat were induced by overweight or obesity.

The cats with a previous CCL injury were significantly older than the sound cats (9.5 ± 1.8 years, and 5.9 ± 3.3 years respectively; P = 0.006). There was no significant difference in weight or BCS between the two groups of cats. There was no correlation between body weight, BCS and any of the gait parameters. Out of the ten cats with CCL injury, six were surgically (S) treated and four were conservatively (C) treated. The surgical method used was the same for five of the cats, the stifle joint being stabilized by lateral retinacular suture technique. In the remaining cat the stifle joint was stabilized with both medial and lateral retinacular sutures. Independently of the chosen treatment, the owners of all cats were advised to keep them in a restricted area, to enforce rest and, for most cats, to try to avoid jumping. Nine out of the ten cats were treated with meloxicam, one cat was treated with tolfenamic acid in connection with the acute injury.

### Pressure mat technique

#### Distribution of the vertical forces in the limbs

Variables from the pressure mat were analysed from two valid trails for each cat. These two trials were selected by looking at video captures and the pressure mat images on the software. The sound cats had a mean velocity of 0.68 (±0.16) m/s and the CCL cats had a mean velocity of 0.66 (±0.16) m/s. On average 10.9 (±2.0) strikes were analysed from the sound cats, and on average 11.4 (±2.8) strikes from the CCL cats. The results of the pressure distribution are presented in Tables 2 and 3. The affected limb is defined as the hindlimb with the predominant findings in the stifle joint at the orthopaedic exam, which is also the limb with the previous CCL injury. The unaffected limb is the hindlimb where the stifle joint has no or minor findings at the orthopaedic examination. The front/hind symmetry index for the variable PVF (%BW) was 1.4 (±0.1) for the cats with a previous CCL injury and 1.2 (±0.1) for the sound cats (P = 0.001). The symmetry index for the same variable on the contralateral front/hind limb pair, that is the limb pair on the opposite the side of the affected hind limb, was 1.68 (±0.29).

**Table 2:** Peak vertical force (PVF) [% body weight (BW)] and vertical impulse (VI) (%BW*sec) for hindlimbs in cats with previous cranial cruciate ligament (CCL) injury and sound cats

Variable	Cats with CCL n = 10 (mean ± SD)	Sound cats n = 15 (mean ± SD)	P value
Peak vertical force (%BW)	22.4 ± 2.4	27.1 ± 3.6	0.0006
Vertical impulse (%BW*sec)	8.1 ± 1.7	8.2 ± 1.5	0.81

Data expressed as mean ± standard deviation (SD)

**Table 3:** Peak vertical force (PVF) [% body weight (BW)] and vertical impulse VI (%BW*sec) for the affected and the unaffected hindlimb of cats with previous CCL injury

Variable	Affected hindlimb n = 10 (mean ± SD)	Unaffected hindlimb n = 10 (mean ± SD)	P value
Peak vertical force (%BW)	21.9 ± 2.4	23.1 ± 2.3	0.0024
Vertical impulse (%BW*sec)	7.6 ± 1.5	8.8 ± 1.7	0.0009

Data expressed as mean ± standard deviation (SD)

#### Distribution of the vertical forces in the paws

**Fig. 2:**
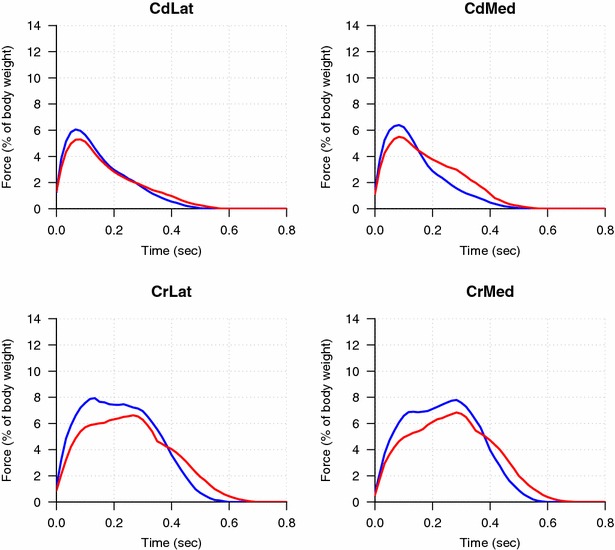
Pressure distribution under the hind paws of 9 cats with a previous CCL injury (*red*) and 15 sound cats (*blue*). Vertical force [% body weight (BW)] vs time (sec) in each of the four quadrants of the hind paws. Craniolateral (CrLat), Caudomedial (CdMed), Craniolateral (CrLat), Craniomedial (CrMed)

The results of pressure distribution within the paws are based on data from nine cats with previous CCL injury. The data from one cat were excluded due to polydactyly. The pressure distribution under the paws of the hindlimbs differed compared to sound cats (Fig. 2). There was no significant difference in VI (%BW). However, cats with a previous CCL injury had a significantly lower PVF (%BW) and a longer duration of stance phase, compared to sound cats. As previously reported, the cats with previous CCL injury increased the force in the vertical plane towards their forelimbs; however, the distribution within the front paws did not change significantly.

### Owner assessment questionnaires

The maximum total score on the owner assessment questionnaire was 50. There was a significant difference in the total score between the cats with a previous CCL injury (9.9 ± 8.7) and the sound cats (1.1 ± 2.5) (P < 0.031).

## Discussion

The hypotheses of the present study were essentially verified. Thus, cats with a previous CCL injury in the stifle joint showed:

- a higher front/hind symmetry index for PVF but not for VI,
- a decreased PVF and VI on the affected compared to the unaffected hindlimb,
- a different way of distributing the pressure under the paws, and
- a difference in behaviour according to the owner´s subjective assessment, compared to sound cats.

The redistribution of weight, from the affected to the unaffected limbs, registered in the present study may have several explanations. The asymmetry may be due to a mechanical factor, with a reduced ability to stabilise the stifle joint and thus a reduced willingness to put load on the affected hindlimb. This has been shown in both cats [27, 30] and dogs [47, 48] with CCL injuries. However, several factors probably contribute to the pressure asymmetry of the cats with previous CCL injury. Instability of the stifle joint, muscle atrophy, altered proprioceptive ability and a modified gait pattern to avoid or minimise pain, are all likely to contribute. This is supported by the results from the clinical- and orthopaedic examination showing pain on manipulation, muscle mass atrophy and decreased joint range of motion. All the cats diagnosed with CCL were diagnosed by a positive drawer test in the stifle joint concerned. Since some cats had findings on the orthopaedic examination and/or radiographic findings in the contralateral stifle joint, it cannot be completely excluded that they had bilateral orthopaedic disease. Although the predominant findings from the orthopaedic exam were consistent with previous CCL injury, they were not always consistent with the radiographic findings. This discrepancy between clinical and radiographic findings is well known regarding OA. A limitation in studying feline osteoarthritis as a naturally occurring disease in privately owned cats, rather than experimentally induced OA, is that the cats are likely to have several joints affected by OA. The pressure mat has a limitation in detecting bilateral changes, but it is likely to detect alterations when looking at the distribution of the load on all four limbs.

Biomechanical disability and physical discomfort are both explanations for the differences in kinetic values, and the significant difference in PVF, however not in VI between the two groups. Our data suggest that cats with a previous CCL injury use the affected limb in such a way as to decrease the peak force, compensating with longer contact times compared to sound. The overall pressure will therefore be similar to that of sound cats, but it will be distributed differently over time. It is a natural reaction in order to reduce pain during the stance phase. This is also a likely explanation to the altered distribution of pressure within the paw. Our study showed a difference in the distribution of the vertical force between the affected and unaffected hindlimbs. Herzog et al. (1993) showed the same result in cats with transected CCL, registering the decrease in the ground reaction force of the affected hindlimb on a force plate [27]. However, in their study, the significant difference in weight distribution disappeared at 16 weeks after surgery. One possible explanation of the somewhat divergent results can be that their results were based on two cats of young age.

The owner assessment questionnaires showed a change in behaviour, with less physical activity compared to sound cats. The result from the questionnaire verifies that the cat owner can contribute with important information in the evaluation of normal and abnormal behaviour. However, further studies are needed to investigate the possibility of distinguishing between different levels of abnormality using the information obtained from the questionnaire.

The recruited cats with previous CCL injury were overweight, assessed by body condition scores. The present study cannot determine if the overweight was caused by, or was a consequence of, the OA. In order to reduce the measurement errors, the weights of the sound cats were matched to the one of the cats with previous CCL injury, since it is unknown if overweight in itself influences the gait pattern.

Currently, radiographic imaging is considered the primary diagnostic instrument for OA in cats, although the results might not be consistent with the findings from orthopaedic examination [3, 13, 14]. As in other species, cats can have radiological features of osteoarthritis and still have a pain-free joint, and it is possible to have osteoarthritis with severe damage to the synovial cartilage with no, or very few, radiographic signs [1, 9]. Our cats were recruited based on their medical history of CCL injury, and with a clinical suspicion of a secondary OA. Since risk factors for OA are old age and previous trauma, there is a possibility that the cats with a previous CCL injury had concurrent orthopaedic conditions that could have interfered with the results of the study. However, this is not likely, since the combined assessment during the study, with clinical and orthopaedic examination, owner assessment questionnaire, pressure mat registrations and radiographs, showed a unanimous result. Consequently, it is likely that the differences in the results of weight distribution and owner assessments are due to the cats’ adaptation to biomechanical disability and/or joint pain caused by previous CCL injury and concomitant OA.

## Conclusions

The present study show that cats with a previous CCL injury have different gait pattern and a difference in owner subjective assessment of behavior compared to sound cats. The cats redistributed their weight from the affected to the unaffected limbs. It is of great importance that further studies are performed to investigate the long term effects of CCL injury on pain and physical dysfunction and its role in the development of OA in cats. Improved assessment tools for chronic pain caused by OA in cats are needed, both to facilitate diagnostics and to evaluate pain relieving treatment.

## Abbreviations

BCS: body condition score
BW: body weight
C: conservative treatment
CCL: cranial cruciate ligament
CdLat: caudolateral
CdMed: caudomedial
CrLat: craniolateral
CrMed: craniomedial
L: left
OA: osteoarthritis
PVF: peak vertical force
R: right
S: surgical treatment
SD: standard deviation
Sec: second
VI: vertical impulse
